# Early experience and assessment of real-world toxicities with mirvetuximab soravtansine in a heavily pretreated patient cohort with ovarian cancer

**DOI:** 10.1016/j.gore.2025.101738

**Published:** 2025-04-05

**Authors:** Susan M. Lang, Filippos Vingopoulos, Isabel Beshar, Sahana Somasegar, Elizabeth V. Adams, Simone A. Sasse, Talayeh Ghezelayagh, Emily Clair McClung, Amer Karam, Oliver Dorigo, Prithvi Mruthyunjaya, Babak Litkouhi

**Affiliations:** a Division of Gynecology Oncology, Department of Obstetrics & Gynecology, Stanford University, 453 Quarry Road, Palo Alto, CA 94304, USA; b Division of Gynecologic Oncology, Department of Obstetrics & Gynecology, Stony Brook Medicine, Stony Brook, NY 11794, USA; c Department of Ophthalmology, Byers Eye Institute at Stanford, 2452 Watson Court, Palo Alto, CA 94303, USA; d Department of Obstetrics & Gynecology, Stanford University, 453 Quarry Road, Palo Alto, CA 94304, USA

## Abstract

**Introduction:**

Mirvetuximab soravtansine (MIRV) recently emerged as a promising therapeutic option for patients with platinum resistant ovarian cancer (PROC). In trials leading up to its approval, several ocular and other toxicities were identified. We report our experience with MIRV in a less-selected “real-world” population.

**Methods:**

A retrospective review of patients with folate-receptor alpha positive, recurrent PROC treated with MIRV between December 2022-April 2024 was performed. Demographic, treatment, and toxicity data were abstracted from the medical record. The primary outcomes of interest were the incidence and nature of MIRV-related toxicities, as well as the clinical response to treatment.

**Results:**

25 patients were analyzed, and 44 % had >3 previous lines of treatment. The median duration of MIRV treatment 4.7 months, and 36 % of patients received more than 6 months of MIRV. Ocular events were common and occurred early, resulting in grade 1, 2, and 3 toxicity (28 %, 20 %, and 16 % respectively). As a result, 36 % of patients had an increased frequency of ophthalmologic care, 48 % required a medication change, and 32 % had dose reduction, but none required MIRV discontinuation. Pneumonitis occurred in 24 % of patients, was the leading cause of treatment discontinuation and may be associated with significant underlying cardiopulmonary comorbidities and pulmonary disease burden.

**Conclusion:**

Our real-world experience with MIRV mirrors previously published data and suggests a benefit may also be seen in a more heavily pretreated population. Toxicities were similar to those previously reported, although our increased incidence of pneumonitis suggests care when treating patients with significant underlying cardiopulmonary comorbidities.

## Highlights

• The antibody drug conjugate, mirvetuximab soravtansine (MIRV), is a promising therapeutic option for platinum resistant ovarian cancer.
• In a heavily pretreated group of platinum resistant patients with ovarian cancer, treatment response and duration of response mirror previously published data, even in patients receiving ≥3 lines of previous therapy.
• Ocular and non-ocular toxicities are common and may require treatment modification and the intensification of ophthalmologic care.
• Pneumonitis occured more commonly than previously reported, and may be associated with underlying cardiopulmonary comorbidity.

## Graphical abstract

**Figure:**
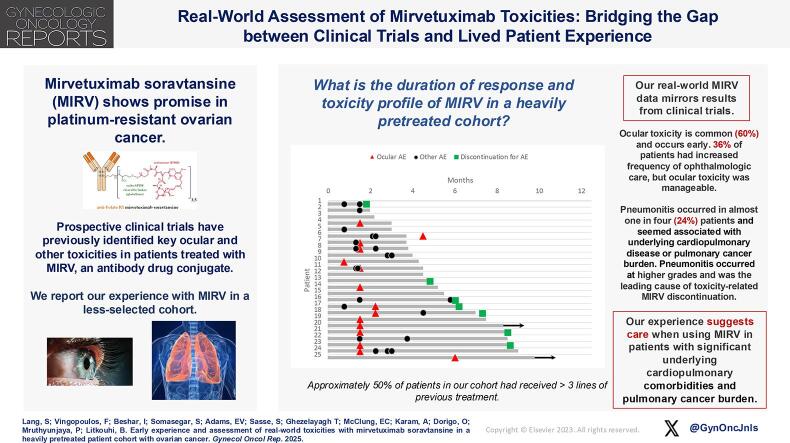

## 1 Introduction

Platinum resistant ovarian cancer (PROC) remains an ongoing therapeutic challenge with response rates to standard chemotherapeutic options ranging from 10-30 %; those who do respond often have a short duration of response in the two to four month range (Poveda et al., 2015, St Laurent and Liu, 2024). Mirvetuximab soravtansine (MIRV), a monoclonal antibody drug conjugate (ADC) targeting folate receptor alpha (FRα) via a microtubule inhibitor, has recently emerged as a promising therapeutic option for patients with PROC with high FRα expression. Based on recent clinical trial data demonstrating objective response rates of 24–42 %, MIRV gained accelerated FDA approval in November 2022 for use in FRα positive, platinum resistant epithelial ovarian, fallopian tube, or primary peritoneal cancer after one to three prior systemic treatment regimens (Matulonis et al., 2025, Moore et al., 2023, Moore et al., 2021). Full FDA approval of MIRV for these indications was granted in March 2024.

Ocular toxicities were amongst the most notable adverse events (AE) in these MIRV trials; these toxicities in fact earned MIRV a black box warning (ImmunoGen, 2024). MIRASOL and SORAYA reported rates of blurred vision (any grade) 41–43 %, keratopathy (any grade) 32–36 %, and dry eye (any grade) 25–28 %, often requiring MIRV dose reduction (up to 34 %), dose delays (up to 54 %) or discontinuation of treatment altogether (up to 9 %) (Matulonis et al., 2025, Moore et al., 2021). Rigorous ocular monitoring was required on trial and is recommended when administering MIRV. Best practice recommendations include a baseline screening eye exam prior to MIRV initiation, with visual acuity and slit-lamp testing, and follow-up eye exams every other cycle for the first 8 cycles and as clinically indicated (ImmunoGen, 2024). Patients are prescribed both lubricating and steroid eye drops for ocular toxicity prevention (ImmunoGen, 2024). Other common AE’s reported include abdominal pain, fatigue, diarrhea, constipation, peripheral neuropathy, neutropenia, anemia, and pneumonitis (Matulonis et al., 2025, Moore et al., 2023).

These clinical trials have provided valuable insight regarding safety profile of MIRV. Rigorous protocols, predefined inclusion and exclusion criteria, and controlled conditions contributed to a systematic assessment of the drug's performance. However, clinical trials do not always broadly represent the general patient population that may derive treatment benefit, as they may exclude certain histologies, patient comorbidities, or heavily pre-treated patient groups. Increasingly, there is a “growing interest in the potential of real-world data to generate fit-for-purpose real-world evidence in cancer care,” and the recent 21st Century Cures Act recognizes the potential for observational evidence to complement higher level data in the drug approval process (Gyawali et al., 2017). Real-world research may expound upon clinical trial data and identify gaps in care (Tang et al., 2023). Differences observed between clinical trials and real-world experiences may reveal a better understanding of the full spectrum of drug toxicities, highlight the impact of comorbidities or longer treatment durations, and further inform management strategies to maximize patient safety and therapeutic outcomes.

After MIRV’s 2022 FDA approval, it was rapidly incorporated into our clinical practice. This manuscript aims to report our experience with regards to toxicities and clinical benefit in a less-selected population after the initial incorporation of MIRV into clinical practice. We hypothesized our experience in this more heavily pretreated population would largely mirror the published prospective trial data.

## 2 Methods

### 2.1 Study design

We performed an Institutional Review Board-approved, retrospective cohort study of patients with FRα positive, recurrent PROC who underwent treatment with MIRV between December 2022 and April 2024. FRα positivity was defined as ≥75 % of viable tumor cells with ≥2+ membranous expression by immunohistochemistry (Matulonis et al., 2025). Testing was done via commercial as well as in-house assays. Participants were identified from the electronic medical record via an institutional database search. We included patients who were older than 18 years of age, received MIRV treatment, and had adequate records within the EMR for review. Patients were included regardless of disease stage, histology or prior treatment history. All patients received routine ophthalmologic care as outlined above and per the manufacturer’s recommendations.

### 2.2 Data collection

Data was extracted for patient demographics, including age, BMI, race, hereditary genetic mutations, number of prior treatment lines, prior bevacizumab exposure, and prior PARP inhibitor exposure. Baseline ECOG performance status and pre-existing cardiopulmonary comorbidities and/or disease burden data were collected. MIRV treatment data were collected, including total number of cycles, dose reductions, treatment delays, and duration of treatment (DOT). DOT was defined as time of treatment start until date of treatment discontinuation recorded in the electronic medical record. MIRV toxicity was also assessed including time to each toxicity, toxicity grade, and changes in treatment due to toxicity. A primary outcome of interest was the incidence and nature of MIRV-related toxicities. We used standard definitions of ACEs as described by the National Cancer Institute’s Common Terminology for Adverse Clinical Events (CTCAE), version 5 (Criteria, 2017). All collected patient information was stored electronically using REDCap (Research Electronic Data Capture) (Harris et al., 2019, Harris et al., 2009).

### 2.3 Statistical analysis

Descriptive statistics were used to summarize data. Categorical variables are presented as percentages, while continuous variables are described using medians as appropriate.

## 3 Results

### 3.1 Study population

Clinical records of 25 patients with recurrent FRα positive PROC treated with MIRV were analyzed. All patients had a baseline performance status of 0 or 1. The median age at treatment initiation was 61 years (range: 45–75) and most patients were non-Hispanic white (64 %). Histologic subtypes included high-grade serous carcinoma in 24 (96 %), and carcinosarcoma in 1 patient (4 %). FRα expression ranged from 75-100 %. Patients had previously received 1 – 7 lines of previous therapy. Eleven patients (44 %) received >3 prior lines of therapy and five (20 %) received >4 prior lines of therapy (6 patients received 4 prior lines of therapy, 3 patients received 5 lines, 1 patient received 6 lines, and 1 patient received 7 lines of therapy, respectively). Baseline characteristics of the patients are listed in Table 1**.**

**Table 1:** Baseline characteristics of MIRV treated patients.

Characteristic	No.	Percent (%)
FRα positive PROC patients	25	
ECOG performance status		
Zero	14	56
One	11	44
Age (years)		
Mean	61	
Range	45–75	
Race/ethnicity		
NHW	16	64
NHB	0	0
Hispanic	2	8
NHAPI	7	28
Home distance to hospital (miles)		
Mean	46	
Range	5–184	
No. prior lines of treatment		
Median	3	
Range	1–7	
> 3 previous lines	11	44
Histology		
High grade serous	24	96
Carcinosarcoma	1	4
Prior bevacizumab		
Yes	23	92
No	2	8
Concurrent bevacizumab		
Yes	1	4
No	24	96
Prior PARPi		
Yes	16	64
No	9	36
BRCA/HRD status		
BRCA1	8	32
BRCA2	0	0
HRD (BRCA wild type)	4	16
HRP	13	52
FOLR1 expression (%)*		
75	5	20
80	6	24
85	3	12
90	3	12
95	3	12
100	4	16
No. of cycles		
Median	7	
Range	2–14	
Duration of treatment (mo.)		
Median	4.7	
Range	2–9.8	
> 6 months	9	36
Ongoing treatment		
Yes	2	8
No	23	92

NHW, Non-Hispanic White; NHB, Non-Hispanic Black; NHAPI, Non-Hispanic Asian and Pacific Islander; PARPi, Poly (ADP-ribose) polymerase inhibitor; HRD, homologous recombination deficient; HRP, homologous recombination proficient; FOLR1, folate receptor alpha.

* 1 data point excluded as no percentage indicated; “High”.

The median number of MIRV treatment cycles was 7 (range: 2 – 14), and median DOT was 4.7 months (range: 2 – 9.8 months). The median DOT for patients having previously received >3 lines of treatment was 5.2 months. A total of 9 patients (36 %) received greater than 6 months of treatment, including 4/11 (36 %) patients having previously received >3 lines of treatment. Two patients (8 %) remained on treatment at the time of data cut-off. Baseline characteristics of the patients are listed in Table 1**.**

### 3.2 Ocular and non-ocular toxicities

Ocular AE were common and are listed in Table 2. Sixty-four percent (16/25) of patients experienced ocular toxicity, 28 % (7/25) with grade 1, 20 % (5/25) with grade 2, and 16 % (4/25) with grade 3. All four patients with grade 3 ocular toxicity had decreased visual acuity/blurry vision. No patients developed grade 4 or 5 ocular toxicity. As a result of ocular AE’s, over one third of patients, 36 % (9/25), required increased frequency of outpatient ophthalmologic care. Only one patient (4 %) had an emergency room visit for ocular concerns. A change in steroid eye drops was necessary for 48 % (12/25) of patients to mitigate ocular toxicities. While ocular toxicity occurred more frequently in patients who had previously received >3 vs ≤3 lines of previous treatment (8/11, 72 % vs 8/14, 57 %), the severity of ocular toxicity in terms of grade distribution was similar.

**Table 2:** Ocular AE of MIRV treated patients.

	No.	Percent (%)
Any ocular toxicity		
Yes	16	64
Grade 1	7	28
Grade 2	5	20
Grade 3	4	16
Grade 4	0	0
Grade 5	0	0
No	9	36
Escalation of outpatient visits		
Yes	9	36
No	16	64
Emergency room visits		
Yes	1	4
No	24	96
Change in steroid drops		
Yes	12	48
No	13	52
Treatment delay		
Yes	5	20
No	20	80
Dose reduction		
Yes	8	32
No	17	68
MIRV discontinued		
Yes	0	0
No	25	100

AE, Adverse events.

Non-ocular AE’s are listed in Table 3. The most common AEs were neuropathy, abdominal pain, constipation, myalgia and pneumonitis. Three patients (12 %) developed grade ≥3 AE, included pneumonitis (n = 2, 8 %) and neuropathy (n = 1, 4 %). As 92 % (23/25) of our patients experienced a non-ocular AE, we were not able to distinguish frequencies between those who had received >3 vs ≤3 lines of previous treatment.

**Table 3:** Non-ocular AE of MIRV treated patients.

	All grade No (%)	Grade 1 No (%)	Grade 2 No (%)	Grade 3 No (%)	Grade 4 No (%)	Grade 5 No (%)
Hematologic						
Anemia	1 (4)	0	1 (4)	0	0	0
Neutropenia	2 (8)	0	2 (8)	0	0	0
Thrombocytopenia	3 (12)	1 (4)	2 (8)	0	0	0
Gastrointestinal						
Abdominal pain	6 (24)	5 (20)	1 (4)	0	0	0
Constipation	5 (20)	5 (20)	0	0	0	0
Diarrhea	4 (16)	2 (8)	2 (8)	0	0	0
Elevated LFT’s	2 (8)	2 (8)	0	0	0	0
Nausea	1 (4)	1 (4)	0	0	0	0
Musculoskeletal						
Fatigue	3 (12)	3 (12)	0	0	0	0
Myalgia	6 (24)	6 (24)	0	0	0	0
Arthralgia	3 (12)	3 (12)	0	0	0	0
Neuropathy	10 (40)	2 (8)	7 (28)	1 (4)	0	0
Pneumonitis	6 (24)	0	4 (16)	1 (4)	0	1 (4)
Treatment delay						
Yes	5 (20 %)					
No	20 (80 %)					
Dose reduction						
Yes	9 (36 %)					
No	16 (64 %)					
MIRV discontinued						
Yes	7 (28 %)					
No	18 (72 %)					

LFT: liver function test.

Table 4 summarizes the baseline cardiopulmonary status of the patients immediately prior to MIRV treatment. Three patients had evidence of moderate to large-sized pleural effusions, while six patients had evidence of hilar/mediastinal lymphadenopathy. No patients had metastatic nodules >2 cm, but two patients had evidence of multiple nodular implants >1 cm. One patient had a previous indwelling pulmonary surgical device (e.g. PleurX), one patients had trapped lung physiology, and one had moderate-to-severe multi-valvular heart disease.

**Table 4:** Baseline cardiopulmonary status immediately prior to initiation of MIRV therapy.

Characteristic	No.	Percent (%)
Moderate-large pleural effusion	3	12
Hilar/mediastinal adenopathy (>1 cm short axis)	6	24
Large > 2 cm parenchymal lung metastases	0	0
Multiple > 1 cm parenchymal lung metastases	2	8
Other significant cardiopulmonary disease*	3	12
Pulmonary surgical device (e.g. PleurX)	1	4

* Moderate to severe COPD, pulmonary hypertension, trapped lung, moderate to severe heart disease.

Six patients were diagnosed with pneumonitis: four patients with grade 2, one patient with grade 3 and one patient with grade 5. All patients presented with dyspnea and/or shortness of breath, and all six were evaluated and co-managed by the Pulmonology service. All six had high-resolution CT chest imaging findings consistent with pneumonitis, with diffuse nodular and ground glass consolidative opacities. Four of the six patient presented to hospital, while two were evaluated and managed as outpatients. Five patients were empirically treated with antibiotics, while additional workup, such as pulmonary function tests or echocardiogram, was performed on an individualized basis. All three patients with moderate to large pleural effusions at MIRV treatment start developed pneumonitis, as did our two patients with trapped lung (one of whom had a PleurX and valvular heart disease). Four of the six patients had one or more of the comorbidities shown in Table 4. Three of these six patients had previously received >3 lines of therapy, while the other three had ≤3 lines of previous treatment.

All six patients diagnosed with pneumonitis were treated with steroids and MIRV discontinuation. Of these six, only one patient received any further chemotherapy; that patient restarted MIRV seven months after resolution of grade 2 pneumonitis, and did not have any of the comorbidities listed in Table 4**.** Five of these 6 patients died between 1 – 8 months after last MIRV treatment, none of whom received any further chemotherapy after MIRV-related pneumonitis. The one pulmonary-related death occurred in a patient with a pre-treatment history of malignant pleural effusions, trapped lung, and mediastinal adenopathy. Upon presentation to hospital, the patient was empirically treated for pneumonia and was also found to have a cytology-proven malignant pericardial effusion resulting in a hemodynamically significant tamponade necessitating pericardiocentesis. In addition, and notwithstanding, CT imaging on presentation was consistent with pneumonitis and steroids were initiated. Given the degree of severity, the Pulmonology service also recommended an additional anti-inflammatory agent, tocilizumab. The patient declined escalation of pulmonary therapy, was ultimately discharged home, and died shortly thereafter (grade 5 AE). The remaining four patients without further therapy had previously received 3, 4, and 5 (N = 2) lines of therapy, and ultimately died from disease progression in the setting of declining performance status.

### 3.3 Dose reductions and delays

Dose reductions, treatment delays, and treatment discontinuation occurred as a result of AE (Table 2, Table 3). Dose reductions due to ocular AE were noted in 32 % (8/25) of patients, while 36 % (9/25) of patients had dose reductions due to non-ocular AE. Treatment delays occurred in 20 % of patients due to ocular AE and non-ocular AE. There were no treatment discontinuations in our population due to ocular AE, although 28 % (7/25) of patients discontinued MIRV due to non-ocular AE. Of these 7 patients, 6 discontinued due to pneumonitis, while one discontinued due to progressive neuropathy.

### 3.4 Toxicity onset

**Fig. 1:**
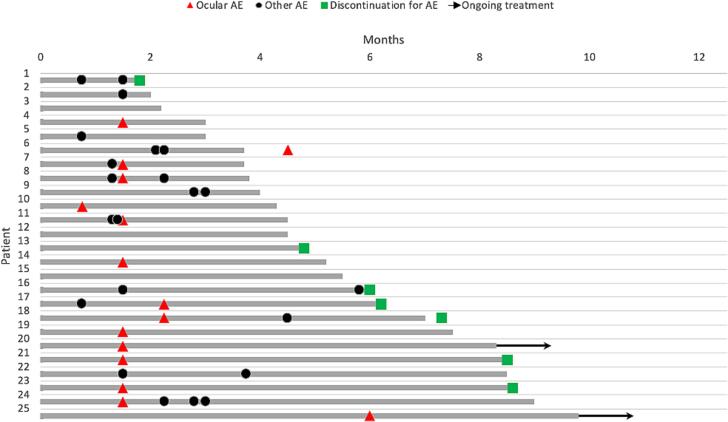
Onset of ocular and other AE in MIRV treated patients. AE, adverse event. Arrows indicate ongoing treatment.

The timing of AE’s varied (Fig. 1). Ocular symptoms and toxicities were most commonly reported and noted between cycle 2 and 3 (69 % of patients). One patient experienced toxicity following the first treatment. Hematologic AE’s, abdominal pain, nausea, diarrhea, and constipation tended to occur in the first two to three cycles, while neuropathy and myalgias were noted throughout the treatment time course. Pneumonitis generally occurred later in the treatment course (cycles 6–12), although in one patient it occurred after after cycle 2.

## 4 Discussion

Several clinical trials have established the clinical benefit of MIRV in the treatment of FRα positive PROC. SORAYA, a single arm phase 2 trial, and MIRASOL, a randomized phase 3 trial comparing MIRV to standard chemotherapy, showed an improved objective response rate, progression free survival, and overall survival compared to standard chemotherapy, with decreased treatment-related and serious AE’s (Matulonis et al., 2025, Moore et al., 2023). The recently reported PICCOLO trial, a single-arm phase 2 trial investigating MIRV in the platinum sensitive recurrent setting, noted promising activity with an overall response rate of 51.9 % (Secord et al., 2024). Ongoing clinical trials (GLORIOSA, NCT05445778) are further investigating the clinical applications of MIRV. This manuscript reports on the real-world experiences associated with MIRV in a less selected and more heavily pretreated population after the initial incorporation of MIRV into our clinical practice.

The key difference in our patient cohort and that of MIRASOL and SORAYA was the heavily pretreated nature of our population. While patients in MIRASOL and SORAYA had received 1–3 prior lines of systemic therapy, the median previous lines of therapy in our patient cohort was 3, with 44 % having received >3 lines. Approximately 15–20 % of patients in MIRASOL and SORAYA had BRCA1/2 mutations, whereas in our patient population, 32 % of patients had a BRCA mutation and 16 % met criteria for homologous recombination deficiency (HRD). Additionally, prior bevacizumab was more prevalent in our patient population and SORAYA (92 % and 100 %, respectively) compared to MIRASOL (61 %). Despite the highly-pretreated nature of our cohort, the median number of MIRV cycles was 7 and median DOT was 4.7 months, which closely mirrors the data reported in MIRASOL (Moore et al., 2023). Notably, these results were not limited to those with less previous therapy, as the median DOT for patients with >3 lines of previous treatment was 5.2 months, with 36 % of these patients receiving greater than 6 months of MIRV. While statistical testing was limited by our small sample size, AE’s seemed to occur at similar frequencies and grades in those who had previously received >3 vs ≤3 lines of previous treatment.

Ocular toxicity is a key treatment related AE associated with MIRV, and in our cohort this seemed to occur at largely similar frequencies and grades in patients who had previously received >3 vs ≤3 lines of treatment. Similar to SORAYA and MIRASOL, the typical onset of ocular toxicities in our patient cohort was after cycle 2 of MIRV (Matulonis et al., 2025, Moore et al., 2023). In these trials, common ocular AE’s included blurred vision (41–43 %), keratopathy (32–36 %), and dry eye (25–28 %) (Moore et al., 2023, Coleman et al., 2024). Our patient cohort also experienced a high (64 %) incidence of ocular toxicities, with 20 % experiencing treatment delay and 32 % having dose reduction; these rates were 53 % and 27 %, respectively, in MIRASOL. Similar to these trials, grade 3 ocular toxicities occurred in 16 % of our patients, although these were limited to decreased visual acuity without keratopathy per se; none of our patients discontinued MIRV secondary to an ocular AE (∼2% in MIRASOL). Nonetheless, 36 % of our patients had increased frequency of ophthalmologic care, 48 % had steroid eye drop adjustment to mitigate ocular AE, and one required emergency department care due to ocular concerns. This may be an important consideration for patients without readily accessible ophthalmologic care; our patient’s average distance to our institution was 46 miles, but ranged from 5 to 183 miles. These data suggest that some patients may face logistical difficulties such as long travel times, high transportation costs, or the need for frequent time off work, all of which can be financially, emotionally, and physically taxing. A randomized phase 2 study of ocular evaluation and toxicity in patients on MIRV is ongoing (NCT06365853).

In MIRASOL, the vast majority of dose reductions and dose delays were due to ocular AE’s, while in our cohort dose reductions and treatment delays due to ocular and non-ocular AE at equal frequencies (about 1/3rd), likely a reflection of the greater cumulative hematologic and neurologic toxicities in our more highly pretreated cohort (Matulonis et al., 2025). On the other hand, similar to MIRASOL and SORAYA, AE-related treatment discontinuations were due to non-ocular toxicities. In terms of non-ocular toxicities, gastrointestinal and hematologic AE occurred at comparatively similar frequencies in our cohort compared to MIRASOL and SORAYA (Matulonis et al., 2025, Moore et al., 2023, Coleman et al., 2024), while our higher 40 % incidence of peripheral neuropathy (MIRASOL, 22 %) again likely reflects a more heavily pretreated population.

Importantly, 24 % of our patients experienced pneumonitis, and this was the leading cause of AE-related treatment discontinuation (six out of the seven patients who discontinued MIRV). Pneumonitis was previously reported in 10 % of patients in SORAYA. It is interesting to note that all three of our patients with baseline moderate to large pleural effusions developed pneumonitis, and that four of the six patients diagnosed with pneumonitis had at least one significant cardiopulmonary comorbidity. Moreover, the two patients who developed grade 3 or higher pneumonitis had significant underlying cardiopulmonary comorbidities, including baseline moderate to large pleural effusions, mediastinal adenopathy and/or moderate to severe valvular heart disease, and previous thoracic surgical interventions. Only one patient with grade 2 pneumonitis and no significant cardiopulmonary comorbidity was able to resume therapy after seven months, while none of the remaining five patients received any further treatment secondary to disease progression after discontinuing MIRV. The one MIRV-related patient death (grade 5 AE) was multifactorial, with pneumonitis considered a likely significant contributing factor.

Notably, investigation of the FDA adverse event system revealed that a number of ADC’s were associated with safety signals for the development of interstitial lung disease, suggesting a potential class effect (Lin et al., 2024). While MIRV was not included in that analysis, a recent case report highlighted the potential for significant pulmonary toxicity from MIRV and reviewed a number of proposed biologic mechanisms for ADC-related pulmonary toxicity (Clark et al., 2024). Both SORAYA and MIRASOL protocols excluded patients with “significant cardiac disease” and a previous history of noninfectious interstitial lung disease (notably, none of our patient cohort met these exclusion criteria). These observations and guidelines collectively suggest consideration when using MIRV in the setting of extensive underlying cardiopulmonary comorbidities and pulmonary cancer burden. It seems reasonable to consider intensified monitoring or screening for the development of drug-induced pneumonitis in such patients, especially as it may be difficult to distinguish symptoms of drug-induced pneumonitis from those of the underlying cardiopulmonary comordibity.

Limitations of this study include the observational nature of this report. We recognize that this study includes a small cohort of patients, and toxicity information was gathered retrospectively from the electronic health records, subjecting it to a number of well-recognized biases. Given the small sample size, descriptive statistics rather than tests of significance were used in the data presentation. Furthermore, given the small cohort, we recognize our observations, including those regarding pneumonitis, should be considered hypothesis generating.

In summary, this report reflects our early real-world experience with MIRV in less-selected patients with PROC. Treatment response and DOT mirror previously published data and suggest a clinical benefit in a more heavily pretreated patient population, almost half of whom had received >3 previous lines of treatment. Toxicities were largely similar to those previously reported, although our experience suggests a frequent need for urgent non-scheduled ophthalmologic care and a similar likelihood of treatment modification for ocular and non-ocular AE’s. Lastly, our experience with pneumonitis suggests careful consideration and surveillance when treating patients with significant underlying cardiopulmonary comorbidities and pulmonary cancer burden.

## Funding Statement

This research did not receive any specific grant from funding agencies in the public, commercial, or not-for-profit sectors.

## Data Sharing Statement

Raw data and data dictionary may be made available upon request.

## Prior presentation

No prior presentation

## Contribution Statement

Lang, Vingopoulos and Litkouhi conceptualized this study. Lang and Beshar conducted data collection. Lang and Beshar drafted the initial manuscript. Lang, Beshar, and Vingopoulos contributed to figures and data curation. Somasegar, Adams, Sasse, Ghezelaygh, McClung, Karam, and Dorigo contributed to manuscript edits. Mruthyunjaya and Litkouhi contributed project supervision and administration.

## CRediT authorship contribution statement

**Susan M. Lang:** Writing – original draft, Formal analysis, Data curation, Conceptualization. **Filippos Vingopoulos:** Writing – review & editing, Data curation, Conceptualization. **Isabel Beshar:** Writing – review & editing, Writing – original draft, Formal analysis, Data curation. **Sahana Somasegar:** Writing – review & editing. **Elizabeth V. Adams:** Writing – review & editing. **Simone Sasse:** Writing – review & editing. **Talayeh Ghezelayagh:** Writing – review & editing. **Emily Clair McClung:** Writing – review & editing. **Amer Karam:** Writing – review & editing. **Oliver Dorigo:** Writing – review & editing. **Prithvi Mruthyunjaya:** Writing – review & editing, Supervision, Conceptualization. **Babak Litkouhi:** Writing – review & editing, Supervision, Project administration, Methodology, Investigation, Conceptualization.

## Declaration of Competing Interest

The authors declare that they have no known competing financial interests or personal relationships that could have appeared to influence the work reported in this paper.
